# Insights into the Mode of Action of Novel Morpholinated Curcumin Derivatives Exhibiting Potent Antitumor Activity in Bladder Cancer Cells In Vitro

**DOI:** 10.3390/molecules30020295

**Published:** 2025-01-13

**Authors:** Paulina Kobylka, Pawel Bakun, Joanna Kuzminska, Tomasz Goslinski, Marek Murias, Malgorzata Kucinska

**Affiliations:** 1 Department of Toxicology, Poznan University of Medical Sciences, Rokietnicka 3 Street, 60-806 Poznan, Poland; paulinakobylka96@gmail.com (P.K.); marek.murias@ump.edu.pl (M.M.); 2 Department of Chemical Technology of Drugs, Poznan University of Medical Sciences, Rokietnicka 3 Street, 60-806 Poznan, Poland; pbakun@ump.edu.pl (P.B.); tomasz.goslinski@ump.edu.pl (T.G.); 3 Department of Pharmaceutical Chemistry, Poznan University of Medical Sciences, Rokietnicka 3 Street, 60-806 Poznan, Poland; jkuzminska@ump.edu.pl

**Keywords:** curcumin, curcumin derivatives, apoptosis, bladder cancer, hypoxia, cancer research

## Abstract

Although curcumin is a well-known natural polyphenol with many biological activities, its clinical application has been limited by low aqueous solubility and stability. Therefore, curcumin derivatives have been proposed to overcome these limitations and increase anticancer activity. This study tested curcumin derivatives with modified feruloyl moieties (**2a** and **2a-B**) and the β-diketo moiety (**2a-B**) to better understand their anticancer mechanism against human bladder cancer cells. The anticancer activity of **2a** and **2a-B** was determined using MTT (hypoxic conditions) and LDH (normoxic conditions) assays. An ELISA-based protein panel was used to find the potential molecular targets, while flow cytometric, colorimetric, fluorescent, and luminescent assays were used to investigate the cell death mechanism. It was shown that compound **2a** exerted a more potent cytotoxic effect under hypoxic conditions, while compound **2a-B** demonstrated a comparable effect in normoxic and hypoxic conditions. The potential molecular targets modified by **2a** and **2a-B** depending on oxygen concentration were also proposed. Both compounds alter cell cycle progression by blocking the cell cycle in the G2/M phase and decreasing the percentage of cells in the G0/G1 phase. Compound **2a-B** led to phosphatidylserine translocation, increased caspase 3/7 activity, and decreased mitochondrial membrane potential, suggesting a mitochondrial apoptosis pathway. We found that the Akt signaling pathway may modulate the activity of compound **2a-B**, as evidenced by enhanced cytotoxic activity in combination with MK-2206, an Akt 1/2/3 inhibitor. Thus, our results provide new insights into the anticancer activity of compounds **2a** and **2a-B**; however, further studies are needed to better understand their therapeutic potential.

## 1. Introduction

Curcumin (1,7-bis[4-hydroxy-3-methoxyphenyl]-1,6-heptadiene-3,5-dione) is a polyphenol derived from the rhizome of the herb *Curcuma longa*. It has demonstrated various biological activities, including antioxidant, anti-inflammatory, antimicrobial, and antiproliferative activities. Due to these activities, curcumin has been considered a potential therapeutic agent for treating several diseases, including cancer and metabolic, cardiovascular, pulmonary, neurologic, and autoimmune diseases [1,2]. The anticancer properties of curcumin are related to multiple signaling pathways such as nuclear factor kappa-light-chain-enhancer of activated B cells (NF-ĸB), phosphatidylinositol 3-kinase (PI3K)/protein kinase B (AKT)/mammalian target of rapamycin (mTOR), Janus kinase/signal transducers and activators of transcription (JAK/STAT), and Wnt/β-catenin, among others [3,4]. From an oncological perspective, curcumin offers several therapeutic benefits, and its anticancer potential can be considered from different perspectives. Firstly, curcumin can act as a chemoprotective agent by inhibiting NF-κB, cyclooxygenases (COXs), lipooxygenases (LOs), signal transducer and activator of transcription 3 (STAT3), xanthine oxidase, and inducible nitric oxide synthase, increasing the antioxidant ability [5,6]; it can also act as a radioprotective agent by decreasing the mRNA expression of fibrogenic cytokines, transforming growth factor (TGF)-β, interleukins (IL-1 IL-6, IL-18), tumor necrosis factor (TNF)-α, and lymph toxin-beta in cutaneous tissues, reducing the expression of cyclooxygenase-2 (COX-2) and NF-κB [6]. Curcumin can also be used as an adjuvant in cancer therapy to sensitize the cells to chemotherapeutic agents and overcome drug resistance [7,8]. Furthermore, curcumin can also be considered a drug candidate that affects the initiation and progression of different cancers by modulating several signaling pathways. Additionally, curcumin is a natural photosensitizer with potential use in photodynamic therapy [9]. However, despite its broad anticancer potential, the clinical application of curcumin in cancer treatment is limited by several factors, including low water solubility, poor stability, insufficient bioactivity in cancer cells, loss of therapeutic efficiency, and lack of selectivity toward tumor cells [10]. Therefore, several approaches have been proposed to improve physicochemical properties and enhance the therapeutic outcomes of curcumin, which can be categorized into chemical and pharmaceutical strategies. Modern medicinal chemistry uses the curcumin scaffold to design novel, more active curcumin derivatives and resolve stability and solubility issues [11,12]. The pharmaceutical strategy focuses on developing formulations, mainly nanoformulations, to increase solubility, enhance stability, and provide tumor-specific delivery. These formulations also allow for the co-administration of curcumin with adjuvants that inhibit or delay its metabolism [13,14].

**Figure 1:**
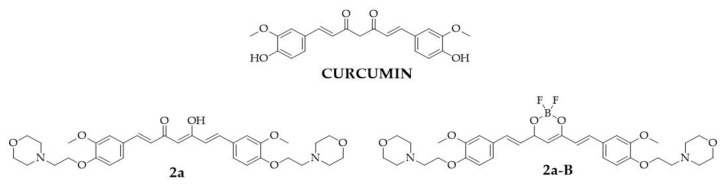
The chemical structures of tested compounds **2a**, **2a-B**, and curcumin, the parental compound.

The curcumin molecule consists of two o-methoxy phenols linked by a seven-carbon chain, forming a heptadiene-3,5-diketone structure [4]. Depending on the environment, the β-diketone moiety in the curcumin structure undergoes keto-enol tautomerization. Its straightforward chemistry, symmetrical structure, and multiple sites for hydrogen replacement make it an attractive target for chemists to synthesize its derivatives [15]. In the structure of curcumin, four main sites can be distinguished: an aryl side chain, a carbon linker, a diketone moiety, and an active methylene moiety, whose modification may improve the effectiveness of curcumin [11]. In this study, we focused on a curcumin derivative with two modifications: the first involving the aryl side chains, where hydroxy groups were replaced by 2-(morpholin-4-yl)ethoxy groups, and the second involving the β-diketo moiety, which was replaced with a BF_2_ group. From a chemical perspective, BF_2_–curcumin complexes can serve as fluorescent dyes since this modification improves fluorescence quantum yields and increases Stokes shifts [16,17]. Furthermore, the presence of the BF_2_ group enhances the rigidity of the 1,3-diketone unit, resulting in improved stability, the shift of the absorption and emission spectra to longer wavelengths, and a reduced nonradiative relaxation rate [18]. From a biological standpoint, BF_2_ insertion may also increase cytotoxicity toward cancer cell lines [19,20,21]. Our previous studies have shown that BF_2_–curcumin complexes, such as fluorinated [22], PEGylated [20], and morpholinated [21] curcumin derivatives, exert a more potent cytotoxic effect than the unmodified β-diketone counterpart. Furthermore, we found that replacing the hydroxy groups in the phenyl ring by the 2-(morpholin-4-yl)ethoxy moiety in the curcumin derivative with both a BF_2_ complex and an unchanged keto-enol moiety increased cytotoxic activity against cancer cells compared to the parental compound (curcumin) [21]. From the series of 30 curcuminoids that were tested in the previous study, two compounds we selected for further investigations: compounds (1*E*,4*Z*,6*E*)-5-hydroxy-1,7-bis({3-methoxy-4-[2-(morpholin-4-yl)ethoxy]phenyl})hepta-1,4,6-trien-3-one (**2a**) and 4-(2-{4-[(1*E*)-2-{2,2-difluoro-6-[(1*E*)-2-{3-methoxy-4-[2-(morpholin-4-yl)ethoxy]phenyl}ethenyl]-1,3,2-dioxaborinin-4-yl}ethenyl]-2-methoxyphenoxy}ethyl)morpholine (**2a-B**). The chemical structures of the tested compounds and the parental compound, curcumin, are presented in Figure 1.

Compounds **2a** and **2a-B** exhibited more potent cytotoxic activity than curcumin against bladder cancer cells. The IC_50_ values for **2a** in the human bladder cancer cell line 5637 were 8.3 µM and 7.5 µM after 24 and 48 h of incubation, respectively. For compound **2a-B**, the cytotoxic activity was further enhanced, with IC_50_ values of 1.2 µM and 1.0 µM after 24 and 48 h, respectively. Given the improved cytotoxic activity of compounds **2a** and **2a-B** compared to curcumin, we conducted further studies to gain more insight into the underlying mechanisms.

## 2. Results

### 2.1. Morpholinated Curcumin Derivatives Exhibited Anticancer Activity Under Hypoxic Conditions

In previous works, we determined the IC_50_ values of curcumin and its derivatives for the human bladder cancer cell lines 5637 and SCaBER under normoxic conditions [21,22]. In this study, we further assessed the activity of tested compounds under hypoxic conditions (Table 1). The dose-response curves are presented in Figure S1. Curcumin derivative **2a** demonstrated increased potency under hypoxia compared to normoxia in both cell lines. For 5637 cells, the IC_50_ value of compound **2a** decreased from 8.3 µM to 5.2 µM, and for SCaBER cells, it decreased from 8.5 µM to 6.2 µM. The IC_50_ values for curcumin and **2a-B** did not change significantly between normoxic and hypoxic conditions. Compound **2a-B** remained the most active under normoxic and hypoxic conditions, with IC_50_ values of 1.2 µM (normoxia) and 1.4 µM (hypoxia) for 5637 cells, and 2.2 µM (normoxia) and 2.4 µM (hypoxia) for SCaBER cells. Regarding curcumin, the parental compound, in our previous study, we showed that curcumin exerted similar activity under both normoxic and hypoxic conditions. For 5637 cells, the IC_50_ values for curcumin were 12.7 ± 3.0 µM and 12.1 ± 0.2 µM for normoxic and hypoxic conditions, respectively [20]. On the other hand, for SCaBER cells, we observed a slightly better effect under hypoxic conditions, with IC_50_ values of 13.1 ± 0.7 µM and 11.6 ± 0.8 µM under normoxic and hypoxic conditions, respectively [20].

The tested compounds exhibited similar activity against 5637 and SCaBER cells, with a slightly lower IC_50_ observed for 5637 cells. Therefore, for further studies, we selected 5637 cells and the most potent compound, **2a-B**, along with compound **2a**, its counterpart without the BF_2_ group.

### 2.2. Curcumin and Its Derivatives ***2a*** and ***2a-B*** Changed the Expression of Stress-Related Proteins Under Normoxic and Hypoxic Conditions

**Figure 2:**
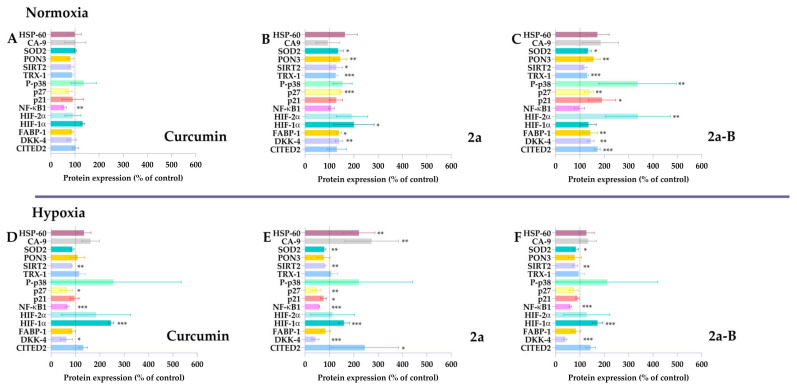
The stress-related proteins modulated by curcumin, **2a**, and **2a-B** under normoxic and hypoxic conditions. 5637 cells were incubated with curcumin (20 µM), **2a** (8 µM), and **2a-B** (2 µM) for 8 h under normoxic conditions (panels (**A**), (**B**), and (**C**), respectively). Similar concentrations and incubation times were used to determine the protein expression under hypoxic conditions; panel (**D**) shows the results for curcumin, while panels (**E**,**F**) show results for compounds **2a** and **2a-B**, respectively. Results are presented as the mean ± SD from two independent experiments. Asterisks indicate statistical significance: * *p* < 0.05, ** *p* < 0.01, *** *p* < 0.001 vs. the control group. Statistical significance was determined using one-way ANOVA with Dunnett’s multiple comparisons tests. Abbreviations: CA9, carbon anhydrase 9; CITED2, Cbp/P300 Interacting Transactivator With Glu/Asp Rich Carboxy-Terminal Domain 2; DKK4, Dickkopf-4; HIF-1, FABP-1, Fatty Acid Binding Protein 1; HSP60, chaperonin 60; Hypoxia-Inducible Factor; P-p38, phosphorylated p38 mitogen-activated protein kinase; p27, Cyclin-dependent kinase inhibitor 1B; p21, cyclin-dependent kinase inhibitor; PON3, paraoxonase 3; SIRT2, sirtuin 2; SOD2, mitochondrial dismutase; TRX-1, thioredoxin-1.

Several curcumin molecular targets have been identified, including enzymes, receptors, growth and transcription factors, and inflammatory cytokines [23]. Depending on the type of cancer cell, curcumin can activate various signaling pathways [23]. Several studies showed that curcumin is a compound capable of inhibiting the progression of bladder cancer cells by triggering apoptosis, inhibiting the PI3K/Akt pathway, blocking the Yes-associated protein (YAP) and/or WW domain–containing transcription regulator 1 (TAZ) pathway, affecting the cell cycle, and suppressing Extracellular signal-regulated kinase 1/2 (Erk1/2) and NF-κB signaling pathways [24]. Studies conducted on the 5637 cell line indicate that treatment with curcumin leads to inhibition of the YAP/TAZ pathway and induction of apoptosis [25]. Moreover, Wu et al. have also demonstrated that curcumin can induce heme oxygenase-1 (HO-1), and using HO-1 inhibitors might increase curcumin efficacy [26]. Understanding the molecular targets and signaling pathways is essential, especially for new compounds. Therefore, in this study, we assessed the impact of the compounds **2a** and **2a-B** on the expression of specific proteins associated with the cellular response to stress factors. The changes in the expression levels of stress-related proteins in treated and untreated cells were analyzed using a proteome profiler, a membrane-based antibody array employing a sandwich immunoassay. Since our results showed that the tested compounds retained their activity under hypoxic conditions, the experiments were performed under normoxic and hypoxic conditions to obtain more insight into the compound’s potential differences in action relating to oxygen concentration. Since each compound was active at different concentrations, we selected the following doses: 20 µM, 8 µM, and 2 µM for curcumin, **2a**, and **2a-B**, respectively. The concentrations of the tested compounds were selected based on the results of the MTT assay, which assessed cell viability after 2, 6, 8, and 24 h of incubation (Figure S2). The chosen concentrations were those that reduced cell viability to 80%, indicating they induced a measurable effect but did not cause strong cytotoxicity or potential degradation of the proteins intended for analysis. The determined IC_20_ values were 8.6 µM for compound **2a** and 1.5 µM for compound **2a-B**. Therefore, concentrations of 8 µM and 2 µM were selected to analyze protein expression for compounds **2a** and **2a-B**, respectively. Protein expression was analyzed after 8 h of incubation, and results are presented in Figure 2 and Figure S3.

Curcumin significantly decreased NF-κB1 expression under normoxic and hypoxic conditions. Notably, NF-κB1 protein expression was the only protein affected by curcumin in normoxia. In contrast, curcumin decreased p27, SIRT2, and DKK-4 under hypoxic conditions. Under these conditions, HIF-1α expression was also significantly increased without significant changes under normoxic conditions.

Compared to curcumin, compound **2a** did not decrease the expression of NF-κB1 in normoxia and affected more proteins than the parent compound. Compound **2a** significantly increased SOD2, PON3, SIRT2, thioredoxin-1, p27, HIF-1α, FABP-1, and DKK-4 expression in 5637 cells. Interestingly, a different pattern was observed for compound **2a** under hypoxic conditions. We found that in hypoxia, **2a** had the opposite effect compared to normoxia on SOD2, SIRT2, p27, and DKK-4, as the expression of these proteins was decreased. Furthermore, NF-κB1 expression significantly decreased under hypoxia but remained unchanged in normoxia. Among the tested proteins, HSP-60, CA-9, CITED-2, and HIF-1α expression increased under hypoxic conditions.

Among the tested compounds, **2a-B** affected the expression of the most proteins compared to curcumin and **2a** under normoxic conditions, suggesting its potential as a broad-spectrum therapeutic agent. Compound **2a-B** significantly increased the expression of several proteins, indicating its potential as a multi-target therapeutic agent. Similar to compound **2a**, NF-κB1 expression in normoxia remained unchanged, suggesting that inserting morpholine groups in feruloyl moiety might reduce the impact on this protein. In contrast, a similar pattern regarding NF-κB1 expression was observed for compound **2a-B** as for **2a** under hypoxic conditions. Compared to results from normoxic conditions, compound **2a-B** under hypoxic conditions decreased the expression of several proteins, suggesting a potential shift in the cellular response to the compound. The only protein whose expression was increased after treatment with **2a-B** in hypoxia was HIF-1α. The expression of proteins that have not changed significantly for tested compounds, such as Bcl-2, cytochrome c, indoleamine-pyrrole 2,3-dioxygenase (IDO), and COX-2, is presented in Supplementary Materials (Figure S3).

### 2.3. Cytotoxic Activity of ***2a*** and ***2a-B*** as a Function of Lactate Dehydrogenase Release from Cells

**Figure 3:**
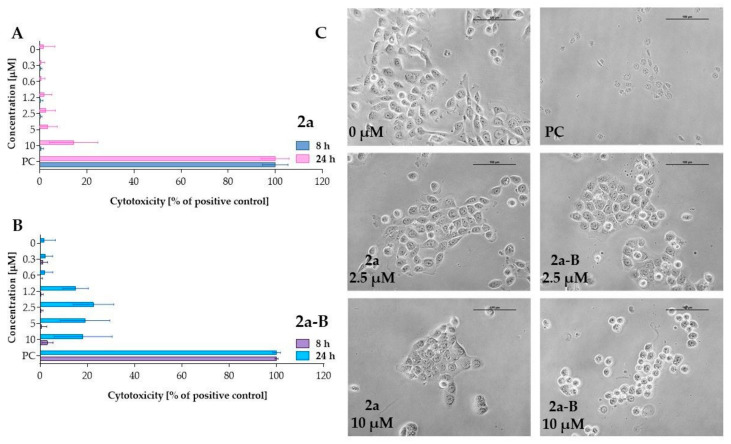
Measurement of LDH release after treating 5637 cells with compounds **2a** and **2a-B**. Panels (**A**,**B**) show the results for compounds **2a** and **2a-B**, respectively. The tested compounds were used at concentrations of 0.3 µM, 0.6 µM, 1.2 µM, 2.5 µM, 5 µM, and 10 µM. LDH release was analyzed after 8 and 24 h of incubation. Panel (**C**) presents representative images of 5637 cells incubated with the tested compounds for 8 h. The photos were taken with a DS-SMc digital camera attached to a Nikon Eclipse TS100 microscope. The scale bar corresponds to 100 µm. Triton X-100 was used as a positive control (PC) at a concentration of 0.9% (*v*/*v*) in the cell culture medium. Data are presented as mean values ± SD from three independent experiments.

The LDH assay assessed the cytotoxic activity of compounds **2a** and **2a-B** (Figure 3). LDH release is a biomarker of cell membrane damage and can indicate both apoptotic and necrotic cell death. The LDH concentration corresponds to the percentage of damaged cells since it is related to loss of membrane integrity. LDH release can be associated with the early stages of necrosis and late stages of apoptosis [27]. After incubation lasting 8 h, both compounds did not significantly affect cell membrane integrity. However, with prolonged incubation time, enzyme release increased for both compounds, and **2a-B** induced increased LDH release starting at a concentration of 1.2 µM. However, the LDH release for the concentrations of 1.2 µM, 2.5 µM, 5 µM, and 10 µM was comparable, indicating that after prolonged incubation at higher concentrations of the compound, there is no concentration-dependent effect. Thus, our results showed that compound **2a-B** can affect cell membrane integrity after incubation lasting 24 h. In contrast, treatment with compound **2a** significantly increased LDH release at a concentration of 10 µM, demonstrating a different cytotoxic profile compared to compound **2a-B**.

### 2.4. Curcumin Derivatives ***2a*** and ***2a-B*** Caused the Changes in Cell Cycle Distribution

**Figure 4:**
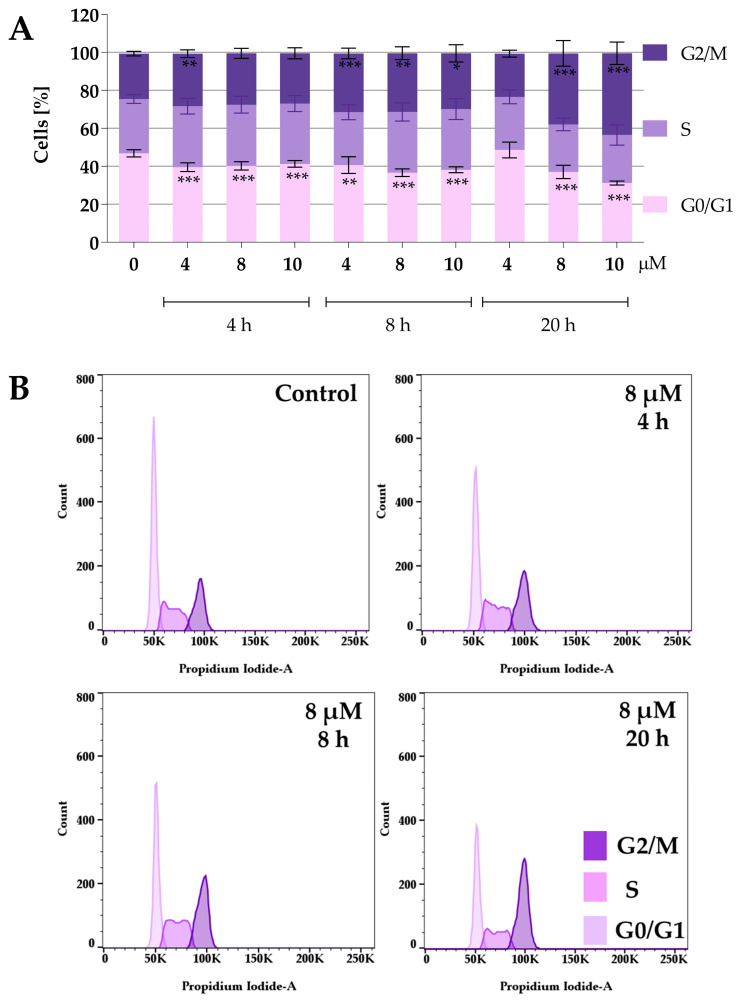
Effect of curcumin derivative **2a** on cell cycle distribution. 5637 cells were treated with compound **2a** at concentrations of 4 µM, 8 µM, and 10 µM for 4, 8, and 20 h. Cell cycle distribution was determined by propidium iodide staining and analyzed using flow cytometry. Panel (**A**) presents the quantitative analysis from three independent experiments, expressed as mean values ± SD. Panel (**B**) presents representative histograms showing cell cycle changes after incubation with compound **2a** at a concentration of 8 µM. Asterisks indicate statistical significance: * *p* < 0.05, ** *p* < 0.01, *** *p* < 0.001 vs. the control group. Statistical significance was measured using one-way ANOVA with Dunnett’s multiple comparisons tests.

**Figure 5:**
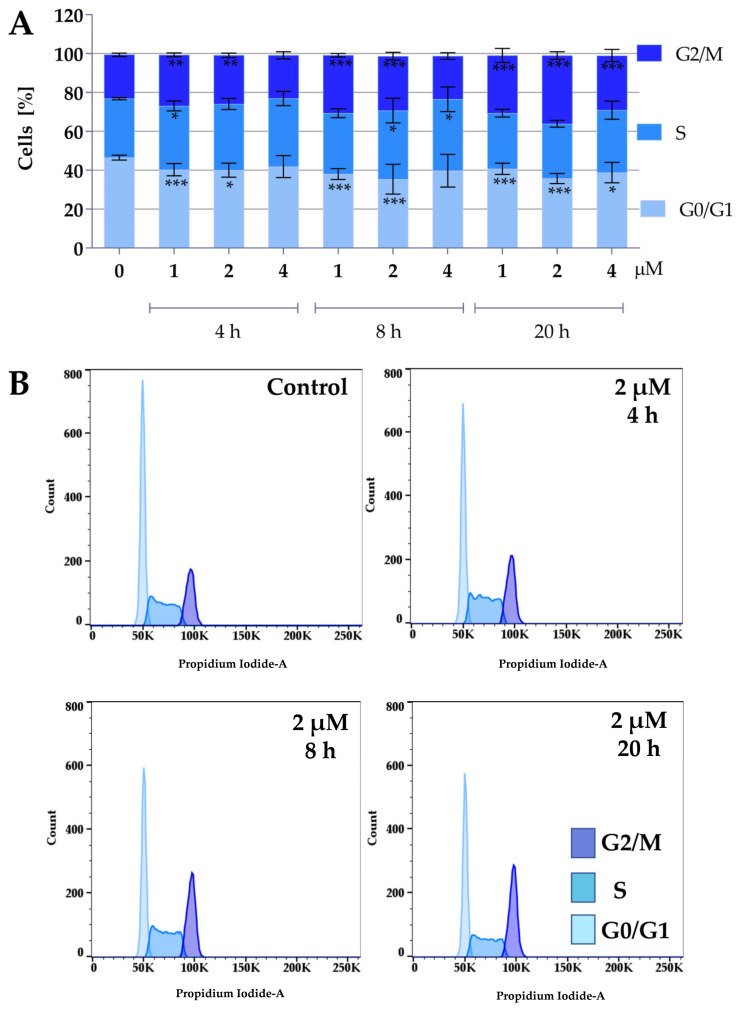
Effect of curcumin derivative **2a-B** on cell cycle distribution. 5637 cells were treated with compounds **2a-B** at concentrations of 1 µM, 2 µM, and 4 µM, respectively, for 4, 8, and 20 h. Cell cycle distribution was determined by propidium iodide staining and analyzed using flow cytometry. Panel (**A**) presents the results as mean ± SD from three independent experiments. Panel (**B**) presents the representative histograms for compound **2a-B** at a concentration of 2 µM. Asterisks indicate statistical significance: * *p* < 0.05, ** *p* < 0.01, *** *p* < 0.001 vs. the control group. Statistical significance was measured using one-way ANOVA with Dunnett’s multiple comparisons tests.

Protein panel analysis revealed that the tested compounds under normoxic conditions caused an increase in the levels of p27 and p21 proteins, indicating that the compounds might affect the cell cycle. Therefore, the following study focused on the effect of the compounds on the cell cycle. To determine the effect of compounds **2a** and **2a-B** on cell cycle distribution, 5637 cells were treated with both compounds at concentrations of 4 µM, 8 µM, and 10 µM for **2a**, and 1 µM, 2 µM, and 4 µM for compound **2a-B**. The concentrations were selected based on the IC_50_ values determined after 24 h and cell viability determined at 2, 6, 8, and 24 h of incubation with the tested compounds. The same concentrations of both compounds were also used for protein expression analysis to identify potential correlations between changes in the cell cycle and specific proteins measured in the previous analysis. Cell cycle distribution was tested after 4, 8, and 20 h of incubation with the tested compounds. The results are presented in Figure 4 and Figure 5.

Both compounds similarly alter cell cycle progression in 5637 cells, mainly blocking the cell cycle in the G2/M phase and decreasing the percentage of cells in the G0/G1 phase. The most potent effect was observed for compound **2a** at concentrations of 8 µM and 10 µM after 20 h of incubation. At a concentration of 8 µM and 10 µM, the cell population in the G2/M phase increased to 37% and 43%, respectively. On the other hand, the maximum accumulation of G2/M in 5637 cells was observed for compound **2a-B** at a concentration of 2 µM after 20 h of treatment (35% of cells). Following **2a** treatment, flow cytometry analysis showed that 5637 cells, after a dose of 8 µM and 8 h of incubation, accumulated in the G2/M phase, with a decreasing number of cells in the G0/G1 phase. Additionally, treatment with **2a** and **2a-B** at 8 µM and 2 µM, respectively, after 8 h of incubation increased the level of p27. These results indicated that **2a** and **2a-B** could arrest bladder cancer cells in the G2/M phase by regulating cell cycle-related proteins, such as p21 and p27.

### 2.5. The Induction of Apoptosis and Necrosis in 5637 Cells After Treatment with Compound ***2a*** and ***2a-B***

Phosphatidylserine (PS) is one of the main phospholipids in the cell membrane, playing a crucial role in maintaining the proper structure of the cell membrane and its functions. PS is a negatively charged phospholipid located on the inner side of the cell membrane [28]. In response to apoptosis or cell activation processes, PS is translocated to the outer part of the cell membrane. During apoptosis, PS translocation is a caspase-dependent process that occurs as an early apoptosis hallmark. However, PS exposure also occurs in non-apoptotic cells during necroptosis [22], pyroptosis [23], or oncosis [29].

**Figure 6:**
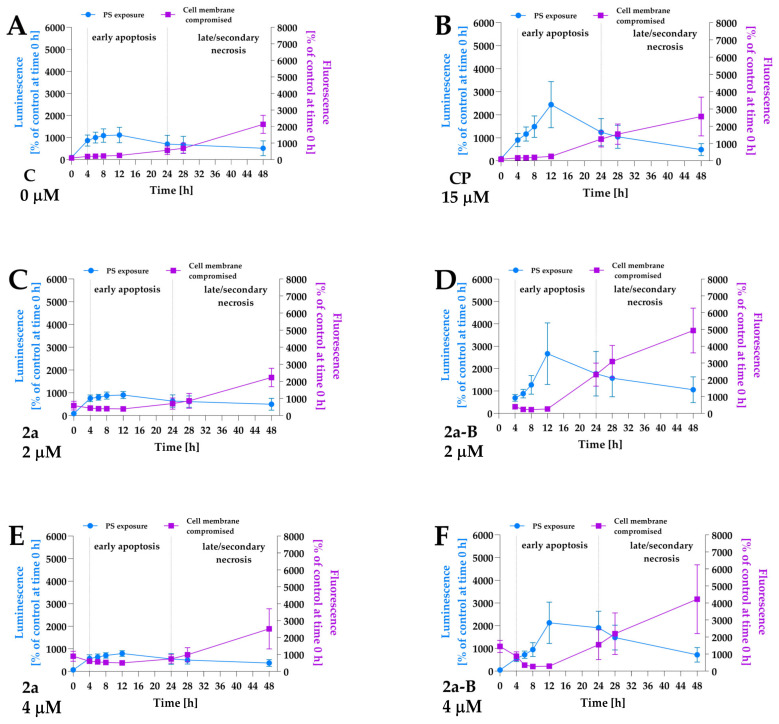
The apoptosis and necrosis/secondary necrosis detection by real-time monitoring of luminescence and fluorescence signals in 5637 cells treated with compounds **2a** and **2a-B**. Panel (**A**) shows the results for control and untreated cells, and panel (**B**) presents the results for the positive control (PC), camptothecin, at a concentration of 15 µM. Panels (**C**,**E**) show the results for compound **2a** at concentrations of 2 and 4 µM, while panels (**D**,**F**) present data for compound **2a-B** at concentrations of 2 and 4 µM, respectively. The measurements were performed for the following time points: 0, 4, 6, 8, 12, 24, 28, and 48 h. The data are presented as mean ± SD from two independent experiments.

To identify the cell death pathways involved in **2a** and **2a-B** anticancer activity, we investigated the apoptosis and necrosis activation in response to compounds **2a** and **2a-B**. In this assay, we used a dye that binds to the PS, indicating apoptotic cells, and a second cell-impermeant, fluorescent DNA dye, which detects necrosis. The assay was performed for compounds **2a** and **2a-B** under normoxic conditions. Since both compounds **2a** and **2a-B** are fluorescent, we initially observed increased fluorescence at the first time point. In the preliminary study, fluorescence values for the tested compounds were measured, and a maximum signal was observed at time point 0 (immediately after adding all reagents), which then decreased over subsequent time points (Figure S4). After 4 h of incubation, the fluorescence signal for the tested compounds reached a value similar to that of the blank (culture medium with reagents used in the assay). Furthermore, a significant increase in fluorescence, indicating secondary necrosis, was observed after 24 h of incubation—a time point at which the fluorescence of the compounds alone was lower than that of the blank and, therefore, could not have influenced the results or the conclusions drawn from these results. Compound **2a** was tested at concentrations of 2 µM, 4 µM, and 8 µM, while compound **2a-B** was tested at concentrations 1 µM, 2 µM, and 4 µM. In this study, we used a concentration of 8 µM for compound **2a** and 1 µM for compound **2a-B**, corresponding to their IC_50_ values after 24 h of incubation. However, we used additional concentrations (2 µM and 4 µM) to better understand the activity of the compounds. Furthermore, to compare the potential pro-apoptotic/necrotic activity between the two compounds, we used the same concentrations (2 µM and 4 µM) for both compounds. Apoptosis and necrosis detection was performed immediately after adding the compounds and reagents and after 4, 8, 12, 24, 28, and 48 h of incubation. Camptothecin at a concentration of 15 µM was used as a positive control to ensure proper data interpretation. The results showed that compound **2a** at all concentrations did not induce apoptosis in 5637 cells (Figure 6 and Figure S5). In contrast, treatment with **2a-B** increased apoptosis, reaching a maximum signal after 12 h at concentrations 1 µM, 2, and 4 µM (Figure 6 and Figure S5), with the strongest increase observed at 2 µM (2666% of control cells measured at time 0). Furthermore, compound **2a-B** exerted a similar effect as a positive control after 12 h of incubation. Interestingly, for concentrations 1 µM and 2 µM after 24 h, we observed an increase in the necrotic cell signal, which overlapped with the luminescent signal. This suggested secondary necrosis (late apoptosis), characterized by increased cell membrane permeability, which can lead to a necrotic-like morphology. These findings are consistent with the LDH release assay (Figure 3), which showed increased LDH levels after 24 h of treatment with compound **2a-B**. Together, these results highlight the dual apoptotic and necrotic/late apoptotic effects of compound **2a-B**, particularly during the extended incubation period.

### 2.6. DNA Fragmentation Detection as a Late Apoptotic Event

**Figure 7:**
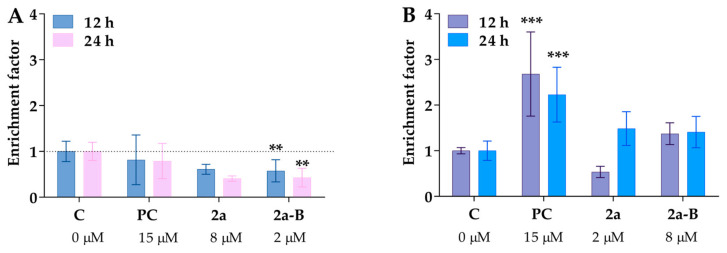
The chromosomal DNA fragmentation is caused by compounds **2a** and **2a-B**. The cytoplasmic histone-associated DNA fragments (mono- and oligonucleosomes) were analyzed in a cell culture medium (necrotic fraction, panel (**A**)) and cell lysate (apoptotic fraction, panel (**B**)). Camptothecin at a concentration of 15 µM served as a positive control (PC) for apoptosis. Asterisks indicate statistical significance: ** *p* < 0.01, *** *p* < 0.001 vs. the control group. Statistical significance was measured using one-way ANOVA with Dunnett’s multiple comparisons tests. Results are presented as mean ± SD from two independent experiments.

An additional assay was used to detect both necrosis and apoptosis induction to obtain more details about the mode of cancer cell death. One of the well-known biochemical hallmarks of apoptosis is the generation of oligo- and mononucleosomes due to chromatin cleavage by endonucleases [27]. Furthermore, a specific form of DNA fragmentation also occurs during necrosis as an early event after cell membrane permeabilization of plasma membranes and is triggered by proteolytic mechanisms different from apoptosis [30]. In this assay, we used concentrations of 8 µM for compound **2a** and 2 µM for compound **2a-B**, consistent with the concentrations used in the protein expression assay and a previous study to monitor apoptosis and necrosis induction. Treatment with compounds **2a** and **2a-B** did not result in the release of DNA fragments to the cell culture medium (Figure 7A). Although both compounds slightly increased DNA fragmentation in 5637 cells, the changes were not statistically significant (Figure 7B). Interestingly, the two compounds exhibited distinct patterns of DNA fragmentation. Compound **2a** increased DNA fragmentation after 24 h of incubation, while compound **2a-B** showed the same effect after 12 and 24 h of incubation. Comparing these results with our previous apoptosis/necrosis detection, it can be concluded that both compounds did not compromise cell membrane integrity, which can be referred to as necrotic-like cell death. Thus, curcumin derivatives seem to activate different cell death modes than necrosis.

### 2.7. The Increased Caspase 3/7 Activity After Treatment with Curcumin Derivatives ***2a*** and ***2a-B***

**Figure 8:**
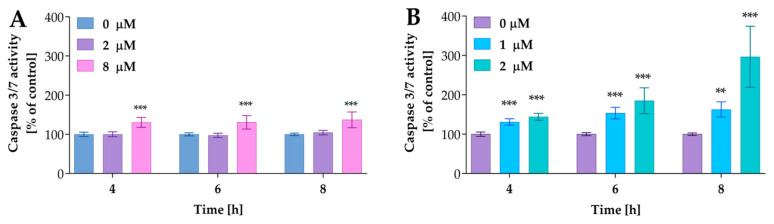
The caspases 3/7 activity after treatment with compounds **2a** and **2a-B**. Panel (**A**) shows the results for compound **2a**, while Panel (**B**) shows the data for compound **2a-B**. Results are presented as mean ± SD from three independent experiments. Asterisks indicate statistical significance: ** *p* < 0.01, *** *p* < 0.001 vs. the control group. Statistical significance was measured using one-way ANOVA with Dunnett’s multiple comparisons tests.

In further studies, we analyzed the activity of caspases 3/7, the key apoptosis executioner. The cells were incubated with compound **2a** at concentrations of 2 µM and 8 µM and **2a-B** at concentrations of 1 µM and 2 µM for 4, 6, and 8 h. The concentrations were selected based on the results from the Real-Time Glo Apoptosis Assay. As presented in Figure 8, compound **2a** increased caspase activity at the highest concentrations; however, this increase was time-independent. On the other hand, compound **2a-B** significantly increased caspase 3/7 activity in a dose- and time-dependent matter.

Compound **2a** increased caspase 3/7 activity by approximately 30% relative to control cells, while compound **2a-B** at a concentration of 2 µM resulted in increases of 44%, 85%, and 196% relative to the control after 4, 6, and 8 h of incubation, respectively. Thus, it can be concluded that caspases are involved in the mechanism of action of **2a-B**.

Furthermore, since caspase 3/7 activity is connected with PS exposure on the outer part of the cell membrane, the significant increase in activity of these enzymes after 8 h correlated with our previous experiments, where we observed the highest increase in apoptosis (as indicated by PS translocation signal) after 12 h, with increasing trends noted after 4, 6, and 8 h of incubation. For compound **2a**, we also observed an increase in caspase 3/7 activity, although this was lower compared to that of compound **2a-B**. As reported by Nano et al., 15 to 30% of cells with intermediate caspase activation led to cell death, and under specific conditions, the apoptotic stimulus can be insufficient to induce cell death via apoptosis [31]. Therefore, apoptosis induction depends on levels or dynamics of active executioner caspases and other factors such as the cellular stress level. Thus, mild executioner caspase activity is not always sufficient to induce apoptotic cell death pathways.

### 2.8. Compounds ***2a*** and ***2a-B*** Change the Mitochondrial Membrane Potential (MMP) of 5637 Cells

**Figure 9:**
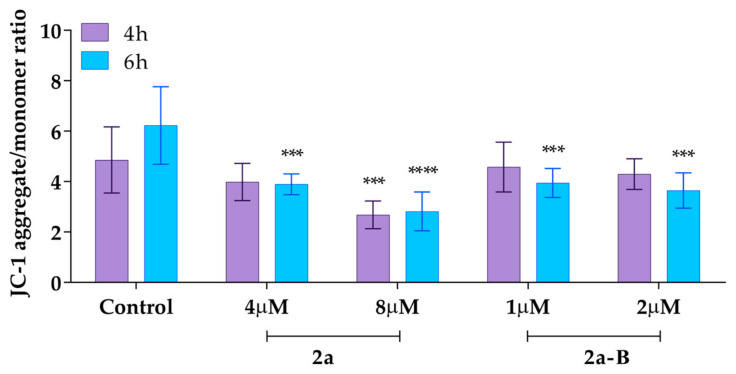
The MMP changes after treatment with compounds **2a** and **2a-B**. Results are presented as mean ± SD from two independent experiments. Asterisks indicate statistical significance: *** *p* < 0.001; **** *p* < 0.0001 vs. the control group. Statistical significance was measured using one-way ANOVA with Dunnett’s multiple comparisons tests.

We also measured the mitochondrial membrane potential using the ratiometric fluorescent probe 5,5,6,6′-tetrachloro-1,1′,3,3′ tetraethylbenzimidazoylcarbocyanine iodide (JC-1). In healthy cells with unaffected MMP, JC-1 dye accumulates in the mitochondria and spontaneously forms reversible complexes called J aggregates, emitting fluorescence at 590 nm [32]. On the other hand, in unhealthy or apoptotic cells, the inside of the mitochondria becomes less negatively charged due to increased membrane permeability and consequent loss of electrochemical potential. Under these conditions, JC-1 cannot reach a sufficient concentration to form J aggregates and emits monomer green fluorescence at 530 nm [32]. Thus, the ratio between aggregates and monomer fluorescence indicates the changes in MMP. In this analysis, the cells were treated with compound **2a** at concentrations of 4 µM and 8 µM, while compound **2a-B** was used at concentrations of 1 µM and 2 µM, as these concentrations were previously used to assess the induction of apoptosis and necrosis in earlier experiments. The results demonstrated that compound **2a** at a dose of 8 µM significantly decreased MMP after 4 and 6 h of incubation to 55% and 45% (compared to the untreated control), respectively (Figure 9).

On the other hand, at a dose of 4 µM, we also observed a decrease in MMP to 82% and 64% compared to the untreated control after 4 and 6 h of incubation, respectively. Compound **2a-B** affected the MMP at both of the tested concentrations only after a longer incubation time. After 6 h of incubation, compound **2a-B** decreased MMP to 63% and 58% compared to the untreated control for doses of 1 µM and 2 µM, respectively.

### 2.9. Oxidative Stress Generation Analysis After Treatment with ***2a*** and ***2a-B***

The results from the stress-related protein array showed that after treatment with **2a** and **2a-B**, the expression of SOD2 and thioredoxin-1 increased. As reported in the literature, the increased oxidative stress may specifically activate SOD2 expression in response to matrix detachment. Trx1 is an important protective cellular antioxidant, and its upregulation protects cancer cells from oxidative stress [33]. Furthermore, thioredoxin-1 levels can increase in multiple cancers, and an increase in Trx1 expression correlates with poor prognosis [34,35,36]. It has been found that the reduced expression of thioredoxin can sensitize bladder cancer T24 cells to doxorubicin, mitomycin C, etoposide, hydrogen peroxide, and UV irradiation [37]. Thus, an analysis of oxidative stress generation by the curcumin derivatives was performed (Figure S6). 5637 cells were exposed to **2a** and **2a-B** at concentrations of 8 µM and 2 µM for 4 and 8 h. For compound **2a**, we did not observe increased oxidative stress. In contrast, after treatment with **2a-B**, oxidative stress slightly increased after 8 h. However, **2a-B** induced only modest oxidative stress generation compared to the positive control. These results suggest that the increase in oxidative stress leads to the activation of antioxidant defense rather than oxidative stress-induced cell death.

### 2.10. The Impact of Curcumin, ***2a***, and ***2a-B*** on Akt Protein

**Figure 10:**
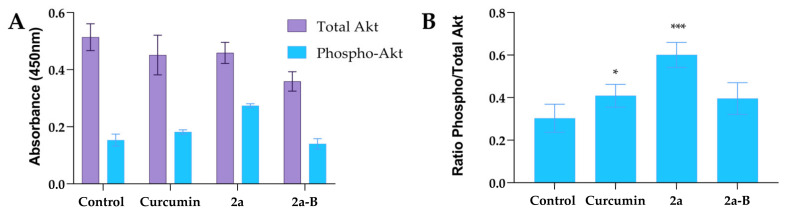
The impact of curcumin and tested compounds **2a** and **2a-B** on the level of Akt protein in 5637 cells. Panel (**A**) shows the effect of tested compounds on total and phosphorylated forms of Akt, while panel (**B**) shows the results expressed as a ratio of phosphorylated form to total Akt. The data are presented as mean ± from two independent experiments. Asterisks indicate statistical significance: * *p* < 0.05, *** *p* < 0.001 vs. the control group. Statistical significance was measured using one-way ANOVA with Dunnett’s multiple comparisons tests.

Based on our previous results, we hypothesized that the Akt pathway could be involved in the mechanism of action of our tested compounds. Thus, we measured total and phosphorylated protein levels using the ELISA method. In the experiments, we used curcumin, compound **2a**, and compound **2a-B** at concentrations of 20 µM, 8 µM, and 2 µM (Figure 10). The protein levels were measured after 8 h of incubation. Interestingly, compound **2a** increased phospho-Akt while compound **2a-B** decreased total Akt and phospho-Akt in 5637 cells.

The ratios of the phosphorylated to total protein level were 0.3, 0.41, 0.60, and 0.39 for the untreated control, curcumin, **2a,** and **2a-B**, respectively. Thus, compound **2a** increased the level of p-Akt two-fold compared to the control cells, while the effects of curcumin and compound **2a-B** were comparable.

### 2.11. The Effect of Combinatorial Treatment Using Curcumin, Curcumin Derivatives ***2a*** and ***2a-B***, and Akt Inhibitor

Since we observed that treatment with both compounds can affect the Akt level differently, we investigated the impact of Akt inhibition on cell viability after treatment with compounds **2a** and **2a-B**. The experiments were also performed for curcumin—the parental compound.

**Figure 11:**
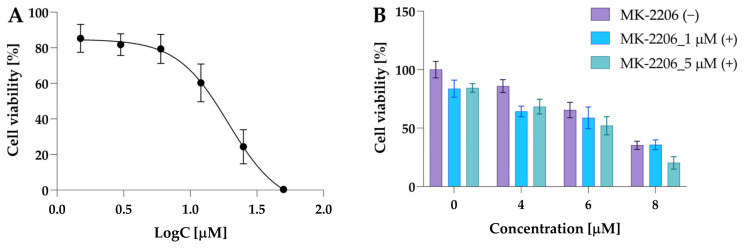
The cytotoxic activity of MK-2206 in 5637 cells. Panel (**A**) shows the dose–response curve used to determine the IC_50_ value. The data are presented as mean ± from three independent experiments. Panel (**B**) shows the results of preliminary experiments performed to select the exact dose of MK-2206. The 5637 cells were incubated with MK-2206, compound **2a**, at concentrations 4 µM, 6 µM, and 8 µM and their combinations. Data are presented as mean ± SD from two experiments.

Firstly, the effect of MK-2206 (8-[4-(1-aminocyclobutyl)phenyl]-9-phenyl-1,2,4-triazolo [3,4-f][1,6]naphthyridin-3(2H)-one dihydrochloride) on 5637 cell viability used in monotherapy was investigated. The 5637 cells were exposed to MK-2206 at a concentration range of 1.5 μM, 3 μM, 6 μM, 12 μM, 25 μM, and 50 μM for 24 h. The IC_50_ value calculated for MK-2206 was 14.9 µM. The dose–response curve for MK-2206 is presented in Figure 11A.

At a concentration of 6 µM, cell viability was 79.37% compared to control cells and 85.3% when MK-2206 was used at a concentration of 1.5 µM (Figure 11B). Based on these results, the preliminary combinatorial treatment was performed using two MK-2206 concentrations: 1 µM and 5 µM. Since compound **2a** increased the p-Akt level and was more potent, this compound was selected for preliminary experiments. The data revealed that both concentrations of MK-2206 similarly decreased cell viability. Cell viability at a dose of 1 µM was 83% of the control and 84% at a concentration of 5 µM, while a potent effect in combination with **2a** was observed for 5 µM of MK-2206 (Figure 11B).

Compound **2a** at a concentration of 8 µM decreased cell viability to 35%, whereas co-treatment with MK-2206 at doses of 1 µM and 5 µM reduced cell viability to 35.7% and 20.3%. Moreover, Jiang and co-workers reported that MK-2206 at a dose of 5 µM effectively decreased Akt phosphorylation in 5637 cells after 15 h of incubation [38]. Consequently, we selected an MK-2206 concentration of 5 µM for further experiments.

**Figure 12:**
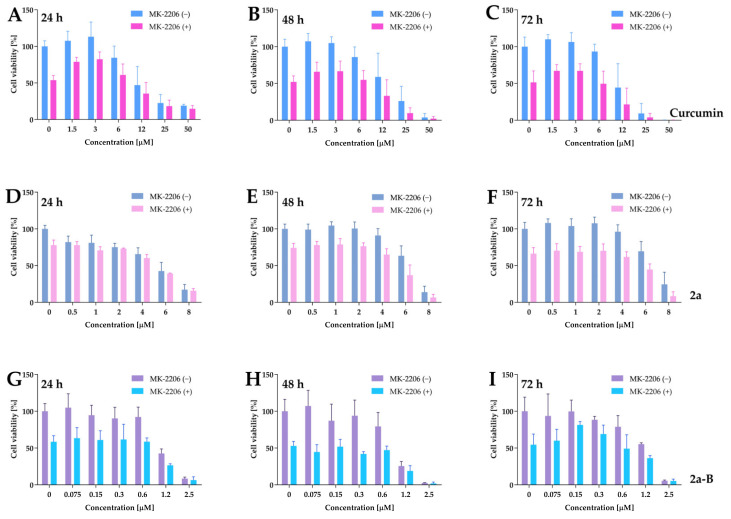
The dose–response curves plotted for curcumin, **2a**, and **2a-B** combined with MK-2206. Panels (**A**–**C**) show the data for curcumin used at concentrations of 50 µM, 25 µM, 12 µM, 6 µM, 3 µM, and 1.5 µM in monotherapy and combination with MK-2206 at a concentration of 5 µM. Panels (**D**–**F**) represent the results for compound **2a**, used in monotherapy at doses of 8 µM, 6 µM, 4 µM, 2 µM, 1 µM, and 0.5 µM and in combination with MK-2206. Panels (**G**–**I**) present the results for compound **2a-B** used at concentration ranges of 2.5 µM, 1.2 µM, 0.6 µM, 0.3 µM, 0.15 µM, and 0.075 µM. For all experiments, MK-2206 was used at a concentration of 5 µM. The effect of monotherapies and combinatorial treatments was determined by MTT assay after 24, 48, and 72 h of incubation. Results are presented as the mean ± SD from three independent experiments.

To further investigate the impact of the Akt inhibitor on the cytotoxic activity of curcumin and its derivatives, 5637 cells were treated simultaneously with curcumin, compounds **2a** and **2a-B**, and MK-2206 for 24, 48, and 72 h. The difference in cell viability between monotherapy and combinations suggests a more additive than synergistic effect (Figure 12).

For example, curcumin at a dose of 6 µM decreased cell viability to 85%, while MK-2206 alone decreased it to 52%, and the combination reduced cell viability to 54%. Similarly, compound **2a** at a concentration of 6 µM reduced cell viability to 63%, and MK-2206 alone to 74%, while their combination reduced cell viability to 37% of the untreated control. Compound **2a-B** at a concentration of 0.6 µM reduced cell viability to 79%, MK-2206 to 52%, and their combination to 47%.

We calculated the IC_50_ values after monotherapy and combined therapy for better interpretation. As presented in Table 2, the treatment with MK-2206 had the most impact on the IC_50_ values of curcumin and **2a-B**. For both compounds, the IC_50_ values for combinations were reduced approximately two-fold compared to single drug use, and this pattern was constant for all time points.

## 3. Discussion

Numerous studies on curcumin and its effects on several crucial cellular processes, including its anticancer properties, have contributed to exploring its health-promoting potential and overcoming the challenges that limit its use as a drug. Thus, various strategies have been proposed to improve its physicochemical properties and pharmacokinetic parameters to enhance curcumin’s efficacy, including using a targeted delivery system, drug combinations, and structure modification. Our research group has modified curcumin to obtain more active compounds for a few years [20,21,22,39]. As a result, we presented two curcumin derivatives with modified aryl side moieties and a seven-carbon linker. Based on our previous studies, we selected two compounds, known as **2a** and **2a-B**, as their effect on cell viability is more potent than that of curcumin.

Hypoxia is a common feature of many solid tumors and is also an adverse prognostic factor in bladder cancer [40]. Since hypoxia is one of the well-known factors that limit the activity of several drugs and drug candidates, we tested the activity of our compound under hypoxic conditions. Our results showed that at the lower oxygen concentration (1% of oxygen), the activity of compound **2a** increased or retained the same effects on 5637 cells (compound **2a-B**). These results align with our previous study on the PEGylated curcumin derivatives. We found that modification in feruloyl moiety with short PEG chains (n = 3) and ether bonds increased cytotoxic activity under hypoxic conditions [41]. The IC_50_ value of compound **5** ((1*E*,4Z,6*E*)-5-hydroxy-1,7-bis(3-methoxy-4-{2-[2-(2-methoxyethoxy)ethoxy]ethoxy}phenyl)-hepta-1,4,6-trien-3-one) against 5637 cells was 8.10 µM, decreasing to 6.13 µM under hypoxic conditions after 24 h, and from 7.91 µM to 5.02 µM after 48 h of incubation. On the other hand, the BF_2_ counterpart exhibited similar activity under both normoxic and hypoxic conditions, with 3.31 µM and 2.58 µM after 24 h and 3.27 µM and 2.96 µM after 48 h, respectively [20]. Similarly, in this study, it was observed that the improvement in activity under hypoxia pertains to curcumin derivatives with modifications in the feruloyl moiety while retaining the unchanged keto-enol group of the seven-carbon linker. 

**Figure 13:**
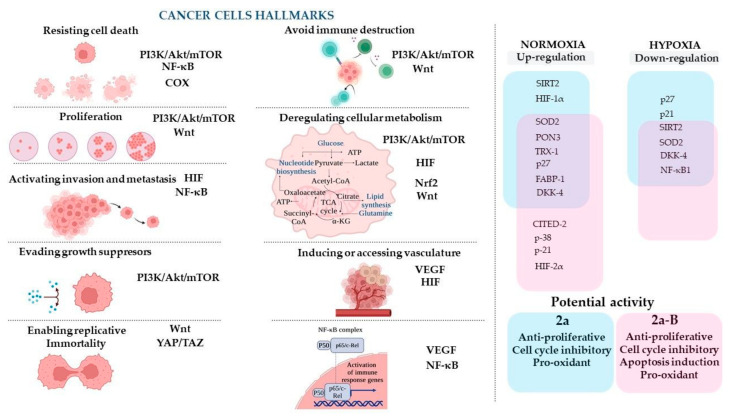
The cancer cell hallmarks concept and proposed molecular targets for compounds **2a** and **2a-B** and their hypothesized anticancer mechanisms. The figure was prepared using BioRender (https://BioRender.com; last accessed on 7 January 2025).

The concept of cancer hallmarks highlights the features/properties of cancer cells that account for the cellular and molecular mechanisms responsible for developing and acquiring abnormal phenotypic capabilities during the initiation and progression of cancer [42]. To discuss the results of the compounds studied and to facilitate the understanding of their potential molecular targets, we decided to refer to those cancer hallmarks and related signaling pathways that may be connected with the expression of the proteins measured in this study (Figure 13).

Based on results obtained from the protein expression analysis panel, we found that compounds **2a** and **2a-B** modulated protein expression differently depending on oxygen concentration. Furthermore, both compounds under normoxic conditions caused an increase in the expression of most of the same proteins, such as SOD2, PON3, TRX-1, p27, FABP-1, DKK-4, and CITED-2. However, it was observed that only compound **2a** significantly increased the levels of proteins SIRT2 and HIF-1α, while compound **2a-B** increased the expression of p38, p21, and HIF-2α. Interestingly, both compounds increased DKK-4 under normoxic conditions. DKK4 proteins can inhibit the activity of the Wnt/β-catenin signaling pathway [43], which involves diverse physiological processes such as proliferation, differentiation, apoptosis, migration, invasion, and tissue homeostasis [44]. Furthermore, the Wnt pathway interacts with other signaling pathways, such as epidermal growth factor receptor (EGFR), Hippo/YAP, NF-κB, Notch, Sonic Hedgehog, and PI3K/Akt pathways [44].

A very interesting result was obtained for the NF-κB1 protein, as only curcumin caused a decrease in its expression under normoxic conditions. The significance of the NF-κB pathway in bladder cancer (BC) is particularly interesting, and its modulation constitutes a promising strategy for increasing the efficacy of BC treatment [45]. NF-κB is involved in innate and adaptive immunity and is a crucial pathway induced during inflammation [23].

Interestingly, we observed that treatment with **2a-B** increased the expression of HIF-2α under normoxic conditions. Jarman et al. reported that HIF-2α may be transcriptionally regulated by HER2 signaling, and HER2 overexpression in MCF-7 cells leads to an increase in HIF-2α but not HIF-1α expression under normoxic conditions [46]. A previous study by Laali et al. showed that fluorinated curcumin–BF_2_ adducts fitted in the tunnel-like binding pocket of HER2, where they mainly established hydrophobic contacts [19]. The authors demonstrated interaction with amino acids similar to the HER2 inhibitor SYR127063 (2-{2-[4-({5-chloro-6-[3-(trifluoromethyl))phenoxy]pyridin-3-yl}amino)-5H-pyrrolo [3,2-d]pyrimidin-5-yl]ethoxy}ethanol), suggesting an inhibitory effect. However, the cytotoxicity studies were conducted on the MDA-MB-231 cell line, which lacks the HER2 receptor. Thus, it is difficult to unequivocally determine whether such interaction with the receptor leads to its inhibition since it may be a more complex antagonistic/agonistic interaction. However, this is interesting because the 5637 cell line is HER2-positive [47,48], and we observed an effect on HIF-2α only for compound **2a-B**, which contains the BF_2_ group, while in compound **2a** with keto-enol moiety, the level of HIF-2α was unchanged.

The observed differences in the expression of certain proteins under normoxic and hypoxic conditions, particularly the reduced levels of proteins such as p21 and p27 observed for compound **2a**, and SIRT2, SOD2, DKK-4, and NF-κB1 observed for both compounds **2a** and **2a-B**, may result from several factors. It is well known that hypoxic conditions can alter cellular metabolism, promote a shift from oxidative phosphorylation to glycolysis [49,50], and reduce ROS generation [49]. Furthermore, hypoxia modulates the expression of multiple genes and proteins to enhance survival under harsh conditions and affects the activity of several signaling pathways [51,52]. Thus, hypoxic conditions can influence the activity of anticancer compounds, as has already been observed for chemotherapeutic drugs [53], radiotherapy [54], immunotherapy [55], and photodynamic therapy [56], leading to decreased therapeutic outcomes. Peixoto et al. showed that 5637 cells incubated in severe hypoxia (0.1% oxygen) exhibit increased levels of glycoproteins, such as integrins and (proto)cadherins, which are involved in cell adhesion [57]. Furthermore, the authors demonstrated that hypoxic conditions enhanced the migration capacity and invasive potential of 5637 cells compared to normoxic conditions, and genetic analysis of 5637 cells suggested possible deregulation in stem cell proliferation and activation of the Wnt signaling pathway [57]. In our experiment, the expression of DKK-4, a key regulator of the Wnt signaling pathway, was significantly modulated: it increased under normoxic conditions and decreased under hypoxic conditions. DKK-4 plays a pivotal role with profound implications for development, cancer, and disease progression. Its dual function in both promoting and inhibiting tumorigenesis underscores its complexity in cellular biology and highlights the need for context-specific therapeutic strategies targeting this protein [58]. Furthermore, our study revealed significant modifications in HIF-1α and SOD2, two essential components involved in cancer cell survival and progression, particularly under conditions of hypoxia and oxidative stress. HIF-1α, a key transcription factor, allows cancer cells to adapt to the hypoxic tumor microenvironment by promoting angiogenesis, metabolic reprogramming, and stress response pathways. This enables tumor cells to survive and thrive in low-oxygen conditions [59]. At the same time, SOD2, a mitochondrial antioxidant enzyme, mitigates oxidative stress by converting harmful superoxide radicals into hydrogen peroxide, which is further detoxified by other enzymes. This process prevents excessive accumulation of reactive oxygen species, thereby protecting cancer cells from apoptosis, autophagy, or senescence [60]. The roles of HIF-1α and SOD2 in cancer are context-dependent. In some tumors, elevated SOD2 activity has been linked to resistance to therapies such as radiation, which depends on ROS-induced damage to kill cancer cells. Conversely, disrupting the functional interplay between HIF-1α and SOD2 could render cancer cells more sensitive to oxidative stress, potentially improving the efficacy of therapeutic interventions [61]. Thus, the mechanism of anticancer activity of compounds **2a** and **2a-B** under hypoxic conditions may differ from that under normoxic conditions. Further studies are needed to clarify how the downregulation of proteins is related to the up- or downregulation of several signaling pathways.

Cyclins and cyclin-dependent kinases (CDKs) are critical for cell cycle regulation. Various types of CDK (1, 2, 3, 4, and 6) and cyclin (A, B, C, D, and E) regulate the cell cycle at different stages. Cyclin C-CDK3 complexes are essential for G0/G1 transition, and complexes of CDK4 or CDK6 with one of the D cyclins (D1, D2, and D3) are involved in the G1 phase, while CDK2/1 complexed with cyclin E or cyclin A is essential for the G1/S transition and DNA synthesis [62]. Complexes between CDK1 and cyclins A and B are required for mitosis [63]. The assembly and catalytic activity of cyclin/CDK complexes are regulated by CDK inhibitors (CDKIs), which can block cyclin–CDK complex activity and prevent cell cycle progression. Two families of CDK inhibitors, INK4 and CIP/KIP, are categorized by their CDK specificity. Members of the INK4 family (p16, p15, p18, and p19) target Cyclin D/CDK4/6, whereas CIP/KIP family members (p21, p27, and p57) can bind all cell cycle-promoting Cyclin/CDK complexes (Cyclin A, B, D, E with respective CDK1, 2, 4, 6) [64].

The CDK inhibitor p21 inhibits cell cycle progression during the G1 and S phases and induces cell cycle arrest in the G2/M phase [65]. Furthermore, p21 reveals multidirectional activity and plays a key role in apoptosis, differentiation, reprogramming of induced pluripotent stem cells, DNA repair, transcription, and cell migration [66]. Another CIP/KIP family member—p27—is a tumor suppressor, inhibiting cell cycle progression and cell proliferation in cancer cells [67,68]. Additionally, p27 also exerts other activities independent of CDK inhibition, such as autophagy, apoptosis, stem/progenitor fate, and cytokinesis [68]. We found that treatment with **2a** and **2a-B** under normoxic conditions significantly increased p27 protein expression. Furthermore, **2a-B** treatment increased p21 expression. Furthermore, we found that under normoxic conditions, compound **2a-B** also increased the expression of p38 protein. The p38 belongs to Mitogen-Activated Protein Kinase (MAPK), which regulates several cellular functions, such as proliferation, differentiation, stress response, apoptosis, migration, and survival [69]. The p38 signaling pathway is also involved in cell cycle regulation, leading to the induction of G1/S cell cycle arrest by phosphorylation and stabilization of p21 and induction of p16 [70]. Moreover, p38-controlled G2/M transition and high p38 activity can prevent G2 progression to mitosis by preventing the activation of Cyclin B/CDK1 [70]. Cell cycle distribution analysis further confirmed the impact on the cell cycle by compounds **2a** and **2a-B**. We found that **2a** caused the G2/M block and decreased G0/G1 at the same doses and incubation time as performed for protein analysis. Also, compound **2a-B** in these conditions resulted in the blocking of G2/M. These results showed that both compounds can affect the cell cycle distribution, further inhibiting cell proliferation.

Further studies were performed to analyze which mode of cell death pathways can be activated in response to treatment with **2a** and **2a-B** and how the structure of both compounds can affect cell fade. The curcumin parental compound can induce apoptosis in 5637 cells [71,72]. Thus, this work focused on necrotic and apoptotic cell death pathways. The LDH release assay shows that treatment with **2a** did not cause a significant loss of cell membrane integrity. For compound **2a-B**, we observed a moderate increase after 24 h of incubation. Monitoring PS exposure on the outer cell membrane, increasing activity of caspase 3/7, and decreasing mitochondrial membrane potential indicated that **2a-B** might activate apoptotic cell death. However, depending on those factors and incubation time, events suggesting secondary necrosis were observed after a longer incubation time with **2a-B**. Interestingly, the **2a** analog without the BF_2_ moiety seems to induce different effects than necrotic and apoptotic cell death. Considering the intermediate caspase 3/7 increase, decreased MMP, and retention of membrane integrity, it is possible that different cell death pathways, e.g., autophagy, can be involved in cell death; however, this hypothesis should be confirmed by more detailed studies.

Based on the obtained results, such as the increased expression of p27 and p38, we hypothesized that the Akt signaling pathway could be one of the potential molecular targets of the studied compounds. Interestingly, compound **2a** increased phospho-Akt (p-Akt) levels, while compound **2a-B** decreased total Akt and phospho-Akt levels in 5637 cells. Since we specifically observed an increase in p-Akt after treatment with **2a**, we aimed to verify if inhibiting the Akt protein could enhance the cytotoxic activity of **2a**. Additionally, given that **2a-B** reduced Akt phosphorylation, we were also interested in whether this reduction could potentiate its activity. Therefore, we conducted combinatorial treatments using an Akt inhibitor (MK-2206) alongside curcumin, **2a**, and **2a-B**. MK-2206 is an allosteric Akt inhibitor that selectively inhibits AKT1 (IC_50_ = 5 nM), AKT2 (IC_50_ = 12 nM), and AKT3 (IC_50_ = 65 nM) [73]. Studies have shown that MK-2206, when combined with anticancer drugs like gemcitabine [74], cisplatin [74], salinomycin, and ruxolitinib [75], exhibits synergistic effects and can potentially improve therapeutic outcomes. Limited studies show the activity of a combination of curcumin and curcumin derivatives with MK-2206. It was reported that MK-2206, by decreasing the expression levels of GRP78 protein, might affect curcumin-mediated GRP78 induction, which finally led to Akt activity decreasing in human nasopharyngeal carcinoma cells [76]. Chen and co-workers showed that curcumin derivative EF24 ((3*E*,5*E*)-3,5-bis[(2-fluorophenyl)methylene]-4-piperidinone combined with MK-2206 induced ER stress, mitochondrial dysfunction, and apoptosis in gastric cancer cells [77]. Additionally, the authors reported that MK-2206 increased ROS levels in a concentration-dependent manner (at high concentrations between 10 and 40 μM), and that ROS generation is unrelated to Akt inhibition. In our study, contrary to the initial hypothesis, combining **2a** with MK-2206 did not significantly affect IC_50_ values but only slightly improved cytotoxic activity. On the other hand, we observed that combining MK-2206 with either curcumin or **2a-B** led to a more potent cytotoxic effect than monotherapy. Thus, combining curcumin and **2a-B** with an Akt inhibitor is an interesting approach that should be considered in further research.

## 4. Materials and Methods

### 4.1. Cell Culture

The 5637 (human urinary grade II carcinoma) and SCaBER (human bladder squamous cell carcinoma) cells were purchased from ATCC (Manassas, VA, USA). The 5637 cells were cultured in Roswell Park Memorial Institute 1640 (RPMI-1640), while SCaBER cells were cultured in Eagle’s Minimum Essential Medium (EMEM), supplemented with 10% (*v*/*v*) FBS, 2 mM l-glutamine, 1% (*v*/*v*), 100 U/mL penicillin, and 10 mg/mL streptomycin. The cells were cultured under standard cell culture conditions (37 °C and 5% CO_2_). For experiments conducted under hypoxic conditions, cells were cultured at 1% O_2_, 5% CO_2_, 94% N_2_, and 37 °C using a Whitley H35 Hypoxystation (Don Whitley, Bingley, UK). Before experiments under hypoxic conditions, the cells and media used in the experiments were transferred to hypoxia for 24 h.

The reagents used for cell culture, such as fetal bovine serum (FBS), penicillin–streptomycin–L-glutamine solution, Dulbecco’s phosphate-buffered saline (DPBS), and trypsin-EDTA, were obtained from Sigma Aldrich (St. Louis, MO, USA). The cell culture media were purchased from Gibco, Thermo Fisher (Waltham, MA, USA).

### 4.2. Tested Compounds

Compounds **2a** and **2a-B** were synthesized at the Department of Chemical Technology of Drugs, Poznan University of Medical Sciences. The synthesis, purity, and characterization of compounds **2a** and **2a-B** were detailed in our previous study [21].

Curcumin was purchased from Fluorochem (Hadfield, UK).

For all experiments, the tested compounds were dissolved in DMSO (Sigma Aldrich, St. Louis, MO, USA) and stored in dark conditions at −20 °C. DMSO was used as a negative control, and its concentration in the culture medium did not exceed 0.1%.

### 4.3. Cell Viability Assay Under Hypoxic Conditions

The cytotoxic activity of compounds **2a** and **2a-B** under hypoxic conditions was tested against 5637 and SCaBER cells. 5637 and SCaBER cells were seeded at densities of 15 × 10^3^ cells and 10 × 10^3^ cells per well, respectively. Following seeding, the plates were placed in a hypoxia station 24 h before the experiment. The cells were treated with compounds **2a** and **2a-B** at concentrations of 0.3 µM, 0.6 µM, 1.2 µM, 2.5 µM, 5 µM, and 10 µM and incubated for 24 h. After incubation, the MTT assay was performed as described in our previous study [20,21]. Absorbance was measured at a wavelength of 570 nm using a plate reader (Biotek Instruments, Elx-800, Winooski, VT, USA). The dose–response curves were fitted using GraphPad Prism 8.0 software. The experiments were performed as three independent experiments.

### 4.4. Human Cell Stress Protein Array

The Proteome Profiler Human Cell Stress Array Kit (R&D Systems, Minneapolis, MN, USA) was used to analyze stress-related proteins. The array utilizes a membrane containing cell stress antibodies in duplicate and internal controls (three reference spots in duplicate and one negative control in duplicate). 5637 cells were seeded in a 6-well plate at a density of 0.4 × 10^6^ cells per well and incubated for 24 h. The plates were incubated in the Hypoxystation for 24 h for hypoxia experiments. The cells were treated with curcumin, **2a**, and **2a-B**, at a concentration of 20 µM, 8 µM, and 2 µM, respectively, for 8 h.

The cells were lysed using a lysis buffer supplemented with protease inhibitors: Aprotinin (Tocris Bioscience, Minneapolis, MN, USA), Leupeptin hemisulfate (Tocris Bioscience, Minneapolis, MN, USA), and Pepstatin A (Tocris Bioscience, Minneapolis, MN, USA). Total protein concentrations were measured using a bicinchoninic acid protein assay (BCA, Thermo Fisher Scientific, USA), with bovine serum albumin used as the protein standard. Equal amounts of protein (150 μg) for all samples were mixed with a cocktail of biotinylated detection antibodies and incubated with array membranes. All subsequent analysis steps were performed according to the manufacturer’s protocol. Chemiluminescence detection was performed using the biotin–streptavidin–horseradish peroxidase method and analyzed using the Invitrogen iBright system (Thermo Fisher, Waltham, MA, USA).

Densitometric analyses of the arrays were performed using the Image Studio Lite 5.2 software (LI-COR Biotechnology, Lincoln, NE, USA). The pixel intensities of each spot were compared to the control (untreated) and are expressed as percentages of the untreated control. Data are presented as mean values from two independent experiments.

### 4.5. LDH Cytotoxicity Assay

The LDH Cytotoxicity Assay Kit (Cayman Chemical Company, Ann Arbor, MI, USA) was used to measure the release of LDH. 5637 cells, at a density of 15 × 10^3^, were seeded in a 96-well plate and incubated overnight. The cells were treated with compounds **2a** and **2a-B** at concentrations of 0.3 µM, 0.6 µM, 1.2 µM, 2.5 µM, 5 µM, and 10 µM for 8 h and 24 h. Both negative (DMSO alone) and positive (0.9% *v*/*v* Triton X-100, Cayman Chemical Company, Ann Arbor, MI, USA) controls were included in the experiment. After treatment, the supernatants were collected and transferred to a 96-well plate. LDH activity was quantified using a colorimetric assay according to the manufacturer’s instructions. Absorbance was measured at a wavelength of 490 nm using a plate reader (Biotek Instruments, Elx-800, Winooski, VT, USA). The experiments were performed in three biological replicates.

### 4.6. Cell Cycle Assay

5637 cells were seeded at a density of 0.4 × 10^6^ per well in a 6-well plate and incubated overnight under standard cell culture conditions. Compound **2a** was added to the cells at concentrations of 2 µM, 4 µM, and 8 µM, while compound **2a-B** was used at concentrations of 1 µM, 2 µM, and 4 µM. The cells were incubated with the tested compounds for 4, 8, and 20 h. Following incubation, the cells were harvested using Trypsin-EDTA and washed twice with cold DPBS. Next, 70% ethanol was added to the cells, and samples were incubated for 30 min at 4 °C. The cells were then washed twice with DPBS and 50 µL of RNase (Sigma Aldrich, St. Louis, MO, USA) at a concentration of 100 µg/mL, and 200 µL of propidium iodide (Sigma Aldrich, St. Louis, MO, USA), at a final concentration of 50 µg/mL, was added. The samples were incubated for 30 min at 37 °C in dark conditions. Samples were analyzed using a BD FACS Lyric (Becton, Dickinson and Company, Franklin Lakes, NJ, USA), and the data were processed using FlowJo 10 (Becton, Dickinson and Company, Franklin Lakes, NJ, USA). The experiments were performed as three independent experiments.

### 4.7. Real-Time Glo Apoptosis and Necrosis Assay

The Real-Time Glo Annexin V Apoptosis and Necrosis Assay (Promega, Madison, WI, USA) was used to analyze the induction of apoptosis and necrosis by compounds **2a** and **2a-B**. 5637 cells were seeded in a white 96-well plate at a density of 15 × 10^3^ cells per well and incubated for 24 h. The reagents were prepared according to the manufacturer’s protocol. The compounds were added to the cells to obtain final concentrations of 2 µM, 4 µM, and 8 µM for compound **2a**, and 1 µM, 2 µM, and 4 µM for compound **2a-B**. Camptothecin (Sigma Aldrich, St. Louis, MO, USA) was used as a positive control at a concentration of 15 µM. The first measurement was taken immediately after adding the compounds using a Tecan microplate reader (Infinite M Plex, Männedorf, Switzerland). Fluorescence was measured at an excitation wavelength of 485 nm and an emission of 525 nm. Subsequent measurements were made at the following time points: 4, 6, 8, 12, 24, 28, and 48 h. The data were analyzed using GraphPad Prism 8.0. The experiments were performed as two independent experiments.

### 4.8. Cell Death Detection ELISA ^Plus^ Assay

The Cell Death Detection ELISA Plus assay (Roche Holding AG, Basel, Switzerland)) was used to determine whether the cells underwent apoptosis or necrosis in the absence and presence of curcumin derivatives at different concentrations. 5637 cells were plated in a 96-well plate at 15 × 10^3^ cells per well and incubated for 24 h in standard cell culture conditions. Compounds **2a** and **2a-B** were added to the cells at concentrations of 8 µM and 2 µM, respectively. Camptothecin (Sigma Aldrich, St. Louis, MO, USA) was used as a positive control at a concentration of 15 µM. The cells were treated with compounds for 12 and 24 h. The supernatant (necrotic fraction) was collected and stored according to the manufacturer’s instructions. The cells were lysed (apoptotic fraction) and incubated for 30 min at room temperature. Subsequent steps were performed following the manufacturer’s protocol. Absorbance was measured at 405 nm using a microplate reader (Infinite M Plex, Tecan, Männedorf, Switzerland). The DNA fragmentation enrichment factor (absorbance of treated cells/absorbance of untreated cells) was calculated according to the manufacturer’s instructions. The experiments were performed as two independent experiments.

### 4.9. Caspase 3/7 Activity Assay

The activity of caspase 3/7 was determined using the Caspase-Glo^®^ 3/7 Assay Kit (Promega, Madison, WI, USA). 5637 cells (at a density of 15 × 10^3^ cells per well) were seeded in a white 96-well plate and incubated for 24 h at 37 °C and 5% CO_2_. The 5637 cells were treated with compound **2a** at concentrations of 2 µM and 8 µM, while compound **2a-B** was used at concentrations of 1 µM and 2 µM. The cells were incubated with the tested compounds for 4, 8, and 12 h. After incubation, the plates were allowed to reach room temperature, and the reagent mixture was added to each well. The plates were mixed on a plate shaker for 30 s at 300–500 rpm and incubated for 30 min in the dark. Luminescence was measured using a Tecan microplate reader (Infinite M Plex, Männedorf, Switzerland). The experiments were performed as three independent experiments.

### 4.10. Mitochondrial Membrane Potential Changes Analysis

The mitochondrial membrane potential (MMP) changes in 5637 cells following treatment with compounds **2a** and **2a-B** were analyzed using the JC-1 Mitochondrial Membrane Potential Assay Kit. 5637 cells were seeded in a black 96-well plate at a density of 15 × 10^3^ cells per well and incubated overnight. Cells were exposed to compound **2a** (4 µM, 8 µM) and compound **2a-B** (1 µM, 2 µM) for 4 and 6 h. Next, 100 µL of culture medium and 10 µL of JC-1 staining solution were added to the cells and incubated for 15 min. The plates were centrifuged at room temperature for 5 min at 400× *g* and washed with Assay Buffer. The washing step was repeated at least three times. Finally, 100 µL of Assay Buffer was added to all wells, and fluorescence was measured using a Tecan plate reader (Infinite M Plex, Männedorf, Switzerland). To detect the J-aggregate form of JC-1, an excitation wavelength of 535 nm and an emission wavelength of 590 nm were used. In contrast, to detect the monomeric form of JC-1, excitation and emission wavelengths of 485 nm and 535 nm, respectively, were used.

### 4.11. Oxidative Stress Detection

5637 cells were seeded at a density of 0.4 × 10^6^ in a 6-well plate and incubated for 24 h at standard cell culture conditions. The tested compounds **2a** and **2a-B** at concentrations of 8 µM and 2 µM, respectively, were added to the cells and incubated for 6 and 8 h. Next, the cells were washed twice with DPBS, trypsinized, and washed twice with DPBS. Subsequently, 50 µL of CellROX^TM^ Deep Red (Thermo Fisher Scientific, USA) at a final concentration of 5 µM was added to each sample and incubated for 30 min at 37 °C. The cumene hydroperoxide was used at a concentration of 4.5 mM. The cells were washed twice with DPBS and resuspended in 200 µL of DPBS. The samples were analyzed using a BD FACS Lyric flow cytometer (Becton, Dickinson and Company, Franklin Lakes, NJ, USA), and the data were analyzed using FlowJo 10 software (Becton, Dickinson and Company, Franklin Lakes, NJ, USA).

### 4.12. Akt Protein Expression ELISA and Combinatorial Treatment with MK-2206

The total and phosphorylated Akt levels were analyzed using the Total/Phospho Multispecies InstantOne^TM^ ELISA kit (Thermo Fisher Scientific, Waltham, MA, USA). 5637 cells were seeded in a 24-well plate at a density of 0.1 × 10^6^ cells per well. After overnight incubation, the cells were treated with curcumin, **2a**, and **2a-B** at concentrations of 20 µM, 8 µM, and 2 µM, respectively, for 8 h. Subsequently, the cells were lysed, and an ELISA assay was performed according to the manufacturer’s protocol. Absorbance was measured at 450 nm using a microplate reader (Tecan Infinite M Plex, Männedorf, Switzerland).

To determine the IC_50_ value of MK-2206 (Selleck Chemicals, Houston, TX, USA), 5637 cells were seeded at a density of 15 × 10^3^ in a 96-well plate. After overnight incubation, the cells were treated with MK-2206 for 24 h at a concentration range of 1.5 μM, 3 μM, 6 μM, 12 μM, 25 μM, and 50 μM. DMSO was used as a negative control. The MTT assay was performed as described in previous studies [20,21]. The experiments were performed as three independent experiments.

For the preliminary combinatorial study, 5637 cells were seeded in 96-well plates at a density of 15 × 10^3^. The experiments included the following groups: cells treated with compound **2a** only at concentrations of 4 μM, 6 μM, and 8 μM; cells treated with MK-2206 alone at concentrations of 1 μM and 5 μM; and cells treated with all compound concentrations in combination with both concentrations of the inhibitor. The experiments were performed as two independent experiments. Based on these results, the MK-2206 concentration of 5 μM was selected for further experiments.

To determine the IC_50_ values of the combinatorial treatment, 5637 cells were seeded at a density of 15 × 10^3^ per well and treated with compound **2a** at final concentrations of 0.3 μM, 0.6 μM, 1.2 μM, 2.5 μM, 5 μM, and 10 μM; compound **2a-B** at final concentrations of 0.075 μM, 0.15 μM, 0.3 μM, 0.6 μM, 1.2 μM, and 2.5 μM; and curcumin at final concentrations of 1.5 μM, 3 μM, 6 μM, 12 μM, 25 μM, and 50 μM. The following experimental groups were included: cells treated with solvent (untreated control), cells treated with the inhibitor alone, cells treated with the compounds alone, and cells treated with a combination of the compounds and the inhibitor. Cell viability was assessed after 24, 48, and 72 h of incubation using the MTT assay [20,21].

## 5. Conclusions

Several studies have investigated the anticancer activity of curcumin on bladder cancer cells, but data regarding the effect of curcumin derivatives in this context remain limited. Our study demonstrates that curcumin derivatives **2a** and **2a-B** significantly reduced the viability of human bladder cancer cells under normoxic and hypoxic conditions, exhibiting stronger activity than the parent compound curcumin. Potential molecular targets were also analyzed. Results from the proteome profiler of protein related to cellular stress revealed that both compounds **2a** and **2a-B** increased the expression of proteins involved in oxidative stress response (such as SOD2, TRX-1, and PON3), cell proliferation, and metabolism (p27, DKK-4, and FABP-1). Additionally, compound **2a** increased the expression of SIRT2 and HIF-1α, while **2a-B** increased the levels of other proteins such as p21, phosphorylated p-38, CITED-2, and HIF-2α. Notably, we demonstrated that the hypoxic conditions changed the pattern of affected proteins, including DKK-4, SIRT2, and p27, being downregulated compared to normoxic conditions. Interestingly, compounds **2a** and **2a-B** did not reduce the expression of NF-κB1 under normal conditions, unlike curcumin. However, we observed a significant reduction in NF-κB1 expression under hypoxic conditions. Furthermore, our results showed that compounds **2a** and **2a-B** induced different cell death pathways. Compound **2a-B** triggered apoptosis in 5637 cells, as evidenced by phosphatidylserine exposure on the cell membrane, increased caspase 3/7 activity, and loss of mitochondrial membrane potential, suggesting the involvement of the mitochondrial apoptosis pathway. In contrast, compound **2a** did not induce apoptosis or necrosis in 5637 cells. The presented data indicate a high probability that the anticancer activity of compound **2a-B** might be associated with the Akt signaling pathway; however, several aspects require additional study.

## Figures and Tables

**Table 1:** The IC_50_ values of compounds **2a** and **2a-B**. The table presents the cytotoxic effect of compounds **2a** and **2a-B** on two bladder cancer cell lines, 5637 and SCaBER, under normoxic and hypoxic conditions. Data obtained in hypoxia were acquired from at least three independent experiments and are expressed as the mean ± SD; the data for compounds **2a** and **2a-B** under normoxic conditions were previously determined and published [21].

	IC_50_ [µM]			
Cell Line	5637		SCaBER	
Compound	Normoxia	Hypoxia	Normoxia	Hypoxia
**2a**	8.3 ± 0.7 [21]	5.2 ± 0.4	8.5 ± 0.7 [21]	6.2 ± 1.0
**2a-B**	1.2 ± 0.4 [21]	1.4 ± 0.2	2.2 ± 0.8 [21]	2.4 ± 0.5

**Table 2:** IC_50_ values for curcumin, **2a**, and **2a-B** towards 5637 cells, used in monotherapy and combinatorial treatment with MK-2206 (Akt inhibitor). Data from at least three independent experiments are expressed as the mean ± SD.

		IC_50_ [µM]	
Compound	Time [h]	MK-2206 (−)	MK-2206 (+)
**Curcumin**	24	14.4 ± 7.6	9.4 ± 3.9
	48	16.8 ± 7.1	7.7 ± 2.3
	72	14.4 ± 6.6	8.2 ± 3.5
**2a**	24	6.7 ± 0.7	5.4 ± 0.9
	48	6.6 ± 0.6	5.3 ± 0.8
	72	6.9 ± 0.8	5.6 ± 0.6
**2a-B**	24	1.1 ± 0	0.6 ± 0.2
	48	0.8 ± 0.4	0.5 ± 0.4
	72	1.2 ± 0.1	0.6 ± 0.4

## Data Availability

The original contributions presented in this study are included in the article/Supplementary Materials. Further inquiries can be directed to the corresponding author.

## Supplementary Materials

The following supporting information can be downloaded at: https://www.mdpi.com/article/10.3390/molecules30020295/s1, Figure S1. Cytotoxic activity under hypoxic conditions; Figure S2. Dose–response curves for compounds **2a** and **2a-B** after 2, 6, 8, and 24 h of incubation; Figure S3. The effect of curcumin and compounds **2a** and **2a-B**; Figure S4. Fluorescence of compounds **2a** and **2a-B** over time after administration to 5637 cells; Figure S5. The effect of compounds **2a** and **2a-B** at concentrations of 8 µM and 1 µM, respectively, on apoptosis and necrosis induction; Figure S6. Oxidative stress generation after treating 5637 cells with compounds **2a**, **2a-B**, and cumene hydroperoxide (CHP) as the positive control.
