# Supplementary material for: Insights into the Mode of Action of Novel Morpholinated Curcumin Derivatives Exhibiting Potent Antitumor Activity in Bladder Cancer Cells In Vitro
Source: Molecules. 2025 Jan 13;30(2):295. doi: 10.3390/molecules30020295 (PMC11767817; doi:10.3390/molecules30020295)
Supplement: Supplementary file 1 [file molecules-30-00295-s001.zip › molecules-3375728-supplementary.pdf]

Insights into the Mode of Action of Novel Morpholinated Curcumin Derivatives Exhibiting Potent Antitumor Activity in Bladder Cancer Cells In Vitro

Paulina Kobylka [1], Pawel Bakun [2], Joanna Kuzminska [3], Tomasz Goslinski [2], Marek Murias [1] and Malgorzata Kucinska [1,*]

[1] Department of Toxicology, Poznan University of Medical Sciences, Rokietnicka 3 Street, 60-806 Poznan, Poland
[2] Department of Chemical Technology of Drugs, Poznan University of Medical Sciences, Rokietnicka 3 Street, 60-806 Poznan, Poland
[3] Department of Pharmaceutical Chemistry, Poznan University of Medical Sciences, Rokietnicka 3 Street, 60-806, Poznan, Poland

* Correspondence: kucinska@ump.edu.pl

SUPPLEMENTARY MATERIALS

Table of contents

1. Cytotoxic activity under hypoxic conditions 2

Figure S1. The dose-response curves for compounds **2a** and **2a-B** against 5637 and SCaBER cells under hypoxic conditions. 2

2. Cytotoxic activity at different time points 3

Figure S2. Dose-response curves for compounds **2a** and **2a-B** after 2, 6, 8, and 24 hours of incubation. 3

3. Stress-related proteins panel 3

Figure S3. The effect of curcumin and compounds **2a** and **2a-B**. 3

4. Apoptosis and necrosis detection 4

Figure S4. Fluorescence of compounds **2a** and **2a-B** over time after administration to 5637 cells. .. 4

Figure S5. The effect of compounds **2a** and **2a-B** at concentrations of 8 μM and 1 μM, respectively, on apoptosis and necrosis induction. 4

5. Oxidative stress generation 5

Figure S6. Oxidative stress generation after treating 5637 cells with compounds **2a**, **2a-B**, and cumene hydroperoxide (CHP) as the positive control. 5

1. Cytotoxic activity under hypoxic conditions

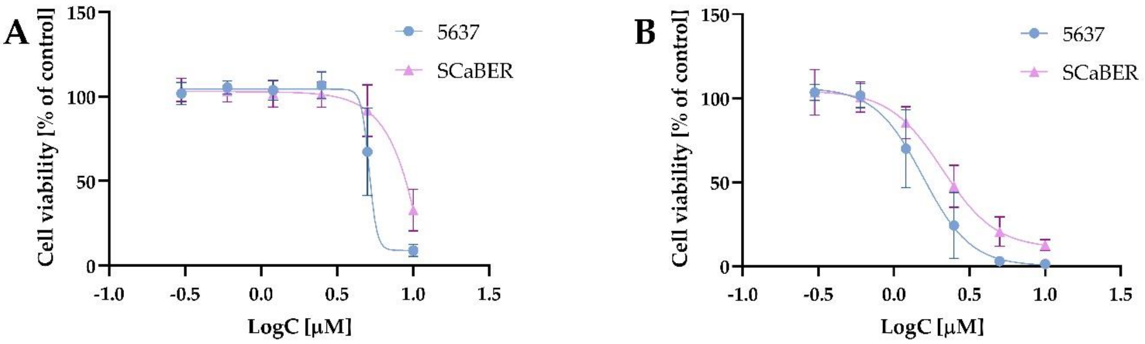

Figure S1. The dose-response curves for compounds **2a** and **2a-B** against 5637 and SCaBER cells under hypoxic conditions. The cell viability was measured using the MTT assay. Panel A shows the data for compound **2a**, while panel B shows the results for compound **2a-B**. The experiments were performed under an oxygen concentration of 1 % using the Hypoxystation, which ensures a constant oxygen concentration. Data are expressed as the mean ± SD from three experiments.

2. Cytotoxic activity at different time points

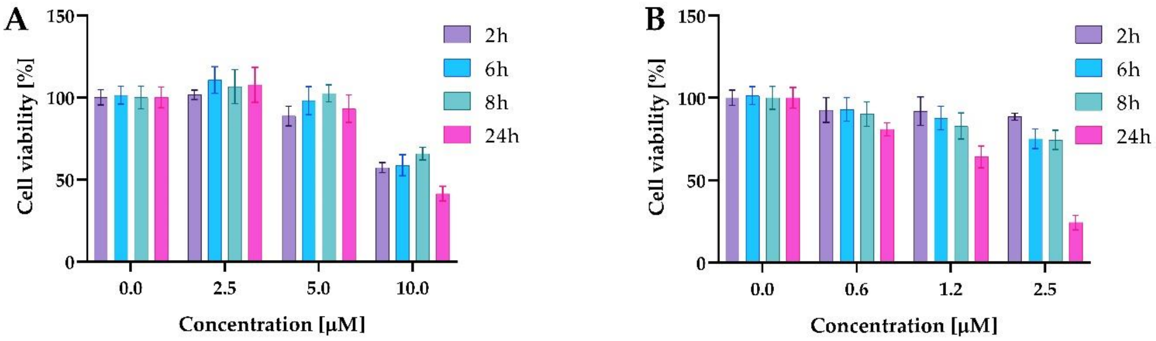

Figure S2. Dose-response curves for compounds **2a** and **2a-B** after 2, 6, 8, and 24 hours of incubation. Cell viability was measured using the MTT assay. Panel A presents data for compound **2a**, while Panel B shows the results for compound **2a-B**. Data are expressed as mean ± SD.

3. Stress-related proteins panel

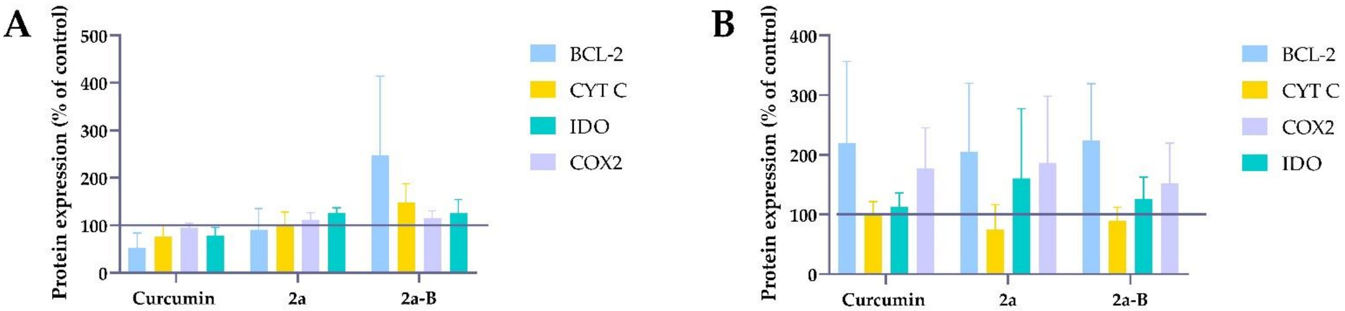

Figure S3. The effect of curcumin and compounds **2a** and **2a-B**. The 5637 cells were treated with curcumin, compounds **2a** and **2a-B** at concentrations of 20 μM, 8 μM, and 2 μM, respectively, for 8 hours. Panel A presents the results for normoxic conditions, while panel B shows the results for hypoxic conditions.

4. Apoptosis and necrosis detection

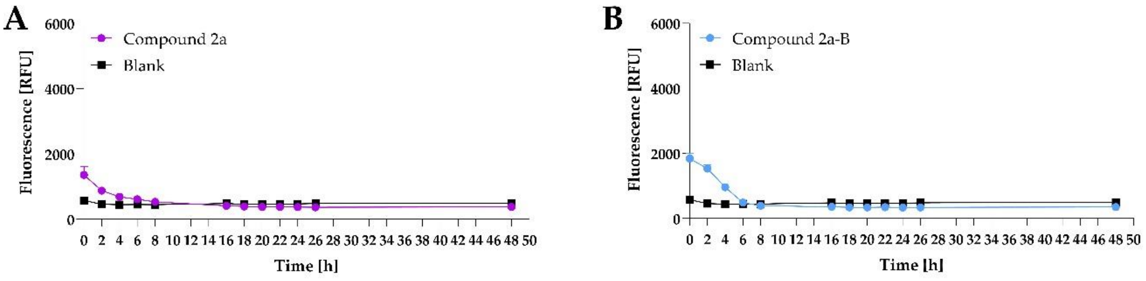

Figure S4. Fluorescence of compounds **2a** and **2a-B** over time after administration to 5637 cells. The analysis was performed to verify the potential signal overlap between the tested compounds and the fluorescent reagent used in this assay. Panel A presents results for compound **2a** at a concentration of 8 μM, while Panel B shows data for compound **2a-B** at the same concentration. Measurements were taken at the following time points: 0, 4, 6, 8, 16, 18, 20, 22, 24, 28, and 48 hours. Fluorescence was measured using a Tecan microplate reader (Infinite M Plex, Männedorf, Switzerland).

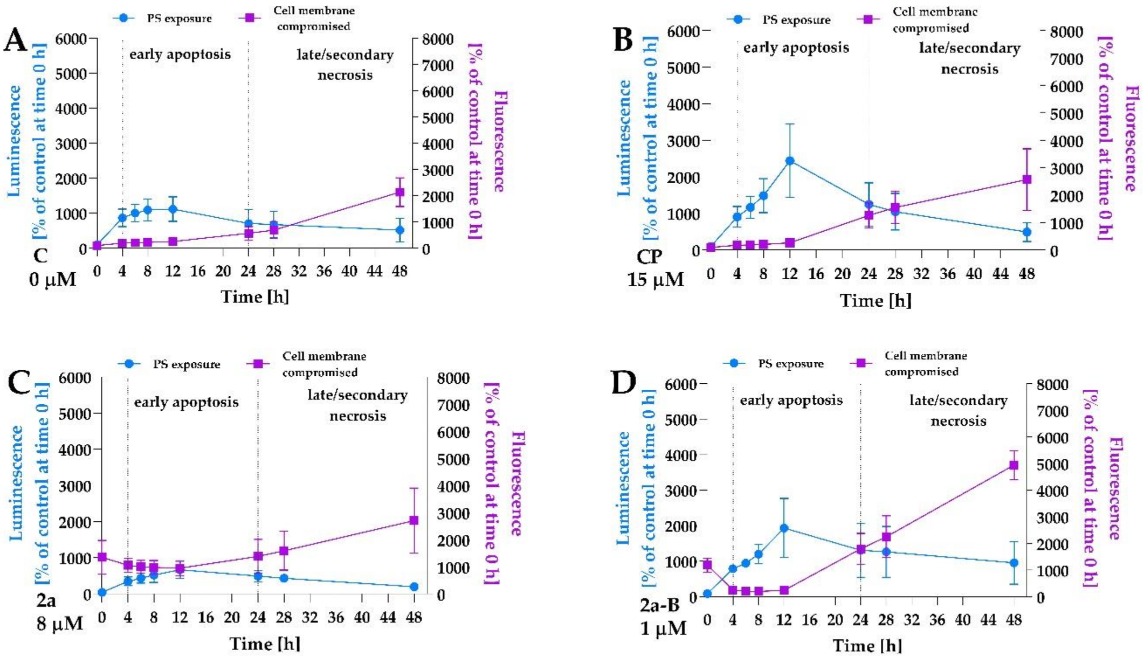

Figure S5. The effect of compounds **2a** and **2a-B** at concentrations of 8 μM and 1 μM, respectively, on apoptosis and necrosis induction. Panel A shows the results for control and untreated cells, and panel B presents the results for positive control (PC) – camptothecin at a concentration of 15 μM. Panel C shows the results for compound **2a** at a concentration of 8 μM, while panel D presents data for compound **2a-B** at a concentration of 1 μM, respectively. The measurements were performed for the following time points: 0, 4, 6, 8, 12, 24, 28, and 48 hours. The luminescence and fluorescence were measured using a Tecan microplate reader (Infinite M Plex, Männedorf, Switzerland).

5. Oxidative stress generation

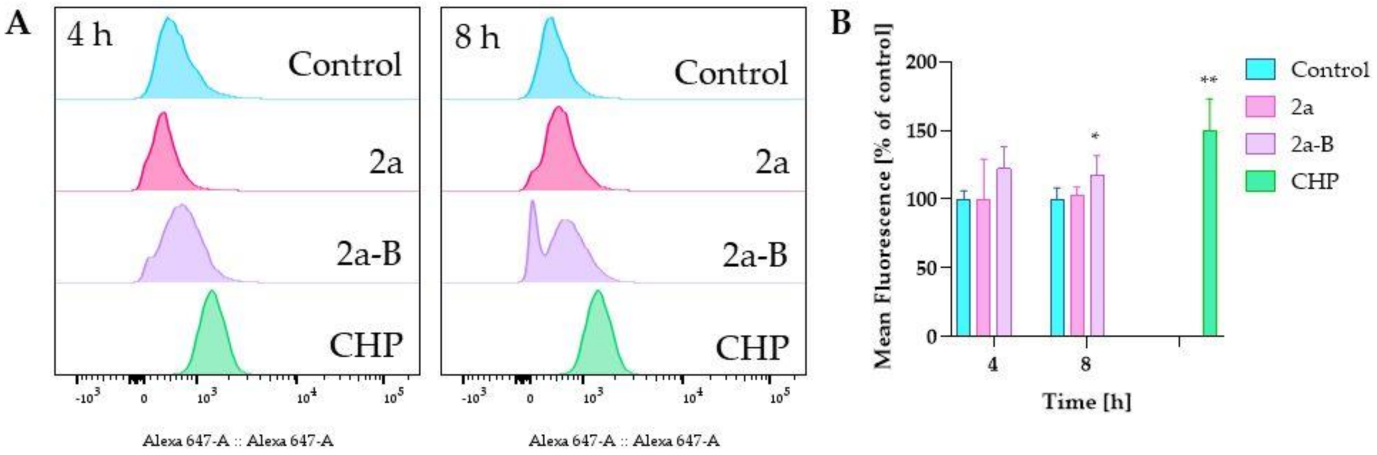

Figure S6. Oxidative stress generation after treating 5637 cells with compounds **2a**, **2a-B**, and cumene hydroperoxide (CHP) as the positive control. Panel A shows representative histograms, while Panel B presents results expressed as mean fluorescence intensity (MFI), shown as a percentage of the control. The graph shows the results from two experiments.
